# Prognostic Factors and Nomogram Construction for First Local Recurrent Retroperitoneal Sarcoma Following Surgical Resection: A Single Asian Cohort of 169 Cases

**DOI:** 10.3389/fonc.2022.856754

**Published:** 2022-04-11

**Authors:** Aobo Zhuang, Weiqi Lu, Yuan Fang, Lijie Ma, Jing Xu, Jiongyuan Wang, Hanxing Tong, Yong Zhang

**Affiliations:** ^1^ Department of General Surgery, South Hospital of the Zhongshan Hospital/Shanghai Public Health Clinical Center, Fudan University, Shanghai, China; ^2^ Department of General Surgery, Zhongshan Hospital, Fudan University, Shanghai, China

**Keywords:** asian, retroperitoneal sarcoma, local recurrent, prognosis, nomogram, recurrence-free survival (RFS), overall survival (OS)

## Abstract

**Objective:**

This study aimed to explore the prognostic factors for first local recurrent retroperitoneal soft tissue sarcoma (FLR-RPS) and construct predictive nomograms in the Asian population.

**Methods:**

In a single Asian sarcoma center, data of patients with FLR-RPS were retrospectively analyzed from January 2011 to September 2020. We developed and internally validated prognostic factors determined by the Cox regression model, as well as nomograms for predicting recurrence-free survival (RFS) and overall survival (OS). The concordance index and calibration curve were used to determine the nomogram’s discriminative and predictive ability.

**Results:**

With 169 patients, the median follow-up duration was 48 months and the 5-year OS rate was 60.9% (95% confidence interval (CI), 51.9%–69.9%). OS was correlated with chemotherapy at the time of initial surgery and tumor grading. The 5-year cumulative local recurrence rate and distant metastasis rate were 75.9% (95% CI, 67.5%–84.3%) and 10.1% (95% CI, 4.2%–16.0%), respectively, and the length of the disease-free interval following the primary operation was associated with disease recurrence. The 6-year OS and cumulative recurrence rate after surgery in our cohort were comparable with those in the TARPSWG cohort, but the proportion of local recurrence was higher (80.4% vs. 59.0%), and distant metastasis was less common (10.1% vs. 14.6%). In this study, two nomogram prediction models were established, which could predict the 1-, 2-, and 5-year OS and RFS, and the concordance indices were 0.74 and 0.70, respectively. The calibration plots were excellent.

**Conclusions:**

For the FLR-RPS patients, some can still achieve an ideal prognosis. The treatment of FLR-RPS in Asian populations can be aided by the predictive model established in this study.

## Introduction

Soft tissue sarcoma is a rare malignant tumor, which accounts for less than 1% of all neoplasm (1). Soft tissue sarcoma that originates in the retroperitoneal space accounts for approximately 15% (2). Even with complete resection, more than half of retroperitoneal sarcoma (RPS) still suffer from local recurrence (LR) within 5 years (3). With prolonged follow-up, the LR rate can reach as high as ~ 90% at 10 years (4) and is the primary cause of mortality (5). Most patients with LR die from small bowel obstruction or cachexia (6, 7). The resectability of recurrent disease is the most important prognostic factor. Specifically, the median survival time for patients with resection of the lesion is 60 months, whereas that with the unresectable disease is only 20 months (8). LR retroperitoneal liposarcoma patients who had monthly growth rates of less than 9 mm and underwent surgical resection had a considerably better prognosis than patients who had monthly growth rates larger than 9 mm, according to Park et al., who focused on first local recurrent RPS (FLR-RPS) (9). Yang et al. reported that the interval between high-grade tumors and disease-free interval (DFI) less than 1 year was a poor prognostic factor for patients with FLR-RPS (10). Similarly, Gronchi and colleagues previously showed that well-differentiated liposarcoma histology subtype and DFI were independent predictors for LR (11). Recently, an Asian sarcoma center published a cohort of 53 patients and discovered that Asian patients with FLR-RPS were younger, had longer DFI, and had a higher tumor burden compared with Western populations. However, due to the limited sample size, it was unable to explore relevant prognostic variables in the Asian population (12).

The nomogram, a predictive tool that incorporates a variety of clinicopathological variables, can effectively be used for individualized treatments. In 2019, Raut et al. reported the outcomes of the largest FLR-RPS set derived from the data of 602 patients with first-time LR of RPS in 22 centers and proposed a new prognostic nomogram (TARPSWG nomogram) using factors including age at second surgery, multifocality, grade, completeness of second surgery, histology, chemotherapy/radiotherapy at first surgery, and the number of organs resected at first surgery (13). To reliably predict the 6-year disease-free survival (DFS) and overall survival (OS) of patients who underwent resection for locally recurrent retroperitoneal sarcoma, the TARPSWG nomogram can be used; however, its research is based on a cohort of European and American populations and without external verification, and it only predicts the patient’s condition at the sixth year after surgery; therefore, its practicality is limited. Moreover, Hui et al. conducted an external validation study on the TARPSWG nomogram using a 53-person Asian cohort. Given the disparities between the Asian cohort and the Western cohort, the concordance indices for 6-year DFS were only 0.65 (12). Therefore, its ability to accurately forecast outcomes in the Asian population needs improvement.

Hence, the purpose of this study is to use the large-sample cohort of a high-flow sarcoma center to explore the prognostic factors of FLR-RPS in the Asian population, as well as to establish prognostic nomograms.

## Methods

### Patient Cohort

Between January 2011 to September 2020 at the South Hospital of the Zhongshan Hospital/Shanghai Public Health Clinical Center, Fudan University, Shanghai, China, 169 patients with curative intent were included. Inclusion criteria were as follows: (1) retroperitoneally located primary tumor, (2) well-differentiated liposarcoma (WDLPS), dedifferentiated liposarcoma (DDLPS), leiomyosarcoma (LMS), malignant peripheral nerve sheath tumor (MPNST), solitary fibrous tumor (SFT), and others (including unclassified/undifferentiated pleomorphic sarcoma, myxoliposarcoma, and pleomorphic liposarcoma) confirmed by pathology (13), (3) no distant metastasis, (4) first relapse, and (5) complete follow-up data. The ethics committee of the South Hospital of Zhongshan Hospital/Shanghai Public Health Clinical Center accepted this study and conducted it in compliance with the Declaration of Helsinki.

The first radical surgical resection of primary RPS was defined as “primary surgery”. “Second surgery” was defined as curative-intent surgery for the first LR. The sum of the diameter of all tumors documented in the surgical record was used to calculate the tumor burden. Complete resection was defined as negative margins (R0) or positive micromargins (R1) but without positive gross margin resection (R2). In accordance with the National Federation of Centers for the Fight Against Cancer (FNCLCC) classification system, all RPS were assigned to one of three grades: grade 1, 2, or 3 (14). DFI was defined as the time from previous operation to the diagnosis of recurrence and was considered a continuous variable.

#### Postoperative Follow-Up

Follow-up data, including clinical and imaging examination results, disease progression, and death were collected (CT or MRI from chest to pelvis). Follow-up examinations were performed every 3 months for 2 years, then every 6 months for 3 years, and once a year after 5 years. The radiographic emergence of a new lesion or considerable expansion of the original lesion was used to indicate disease recurrence.

### Statistical Analysis

Recurrences inside the ipsilateral retroperitoneum, peritoneal cavity, or pelvis were categorized as LR. The second postoperative LR, distant metastasis (DM), or death without recurrence were all defined as the end-point event of DFS, whereas the time between the second surgery to the second recurrence of the disease is classified as recurrence-free survival (RFS). LR combined with DM was recorded as a DM event. Disease recurrence included LR and DM. The survival curve was estimated using the Kaplan–Meier method, and the log-rank test was performed to determine the statistical significance between groups. The crude cumulative incidence curves for LR and DM were calculated in the competitive risk framework. In the analysis of LR (DM), deaths without evidence of disease were considered to be concurrent occurrences with DM (LR). The advancement of the residual tumor following surgery was classified as a LR in the subgroup of patients who underwent *R* ^2^ resection.

Univariate Cox proportional hazards analysis was used to examine the impact of various clinicopathological parameters on survival and recurrence, and variables (*p* < 0.05) were further incorporated into the multivariate Cox model. As for variable selection, all clinicopathological variables were included, and a backward procedure based on the Akaike Information Criterion was applied. It was developed using a multivariate Cox model that included the chosen variable. Discrimination was measured using Harrell C’s consistency index (15). An estimated survival probability was used to split the patients into four groups for calibration. This graph was created by calculating a mean and 95% confidence interval for the survival probability of each subgroup before creating it.

All tests were two-tailed, and a *p* < 0.05 was considered statistically significant. All data were analyzed using the SPSS 22.0 (SPSS Inc., Chicago, IL, USA) and R 4.0.4 (R Foundation for Statistical Computing, Vienna, Austria; http://www.r-project.org/).

## Results

### Patient and Tumor Characteristics

The median follow-up time was 48 (range, 0.3–120) months. Patient characteristics are listed in **Tables 1** and **2**. Sixty-three (37.3%), 50 (29.6%), 29 (17.2%), 6 (3.6%), 4 (2.4%), and 17 (10.1%) cases were diagnosed with WDLPS, DDLPS, LMS, MPNST, SFT, and others, respectively. According to the FNCLCC classification, 60 (35.5%) cases were defined as grade 1, 59 (34.9%) as grade 2, and 50 (29.6%) as grade 3 tumors (**Table 2**). For primary surgery, the median age was 51 (range, 24–82) years, 7 (4.1%) received laparoscopic surgery, and most patients had complete resection (95.9%). The median number of combined resections was 0 (range, 0–5). The median DFI after primary surgery was 14 (range, 0.5–341.9) months (**Table 1**). For second surgery, the median age was 54 (range, 26–84) years. Most patients (94.7%) with complete resection had a median number of combined resections of 2 (range, 0–6). The median tumor burden was 15 (range, 2–55) cm and 44 (26.0%) had multifocal diseases. Twenty-one (12.4%) patients received external beam radiation therapy, and 36 (21.3%) received chemotherapy. The median estimated blood loss was 500 (range, 10–4,500) ml. Intraoperatively packed RBC transfusion was administered in 63 (37.3%) patients, with a median transfusion of 4 (range, 2–14) units. Major postoperative complications occurred in 21 (12.4%) patients, and 5 (2.9%) patients died within 90 days after surgery. The median postoperative hospital stay of all patients was 16 (range, 7–208) days (**Table 2**).

**Table 1:** Patient and tumor characteristics in 169 patients with primary surgery.

Characteristics	*N* = 169	% of total
Age [years, median (range)]	51	24–82
Operation		
Laparoscopic surgery	7	4.1
Open surgery	162	95.9
Complete resection		
Yes	159	94.1
No	10	5.9
Number of combined resections [median (range)]	0	0–5
Radiation		
Yes	5	3.0
No	164	97.0
Chemotherapy		
Yes	17	10.1
No	152	89.9
DFI after first surgery [median (range)]	14	0.5–341.9

DFI, disease-free interval.

**Table 2:** Patient and tumor characteristics in 169 patients with second surgery.

Characteristics	*N* = 169	% of total
Gender		
Men	65	38.5
Women	104	61.5
Age [years, median (range)]	54	26–84
ASA score		
1	130	76.9
>1	39	23.1
Operation		
Laparoscopic surgery	0	0
Open surgery	169	100
Complete resection		
Yes	160	94.7
No	9	5.3
Number of combined resections [median (range)]	2	0–6
Tumor burden [cm, median ](range)	15	2–55
Histologic subtypes		
WDLPS	63	37.3
DDLPS	50	29.6
LMS	29	17.2
SFT	4	2.4
MPNST	6	3.6
Other	17	10.1
Multifocality		
No	125	74.0
Yes	44	26.0
FNCLCC		
Grade 1	60	35.5
Grade 2	59	34.9
Grade 3	50	29.6
Radiation		
Yes	21	12.4
No	148	87.6
Chemotherapy		
Yes	36	21.3
No	133	78.7
Operative time [h, median (range)]	4	1–12
Estimated blood loss [ml, median (range)]	500	10–4,500
Packed RBC transfusion		
Yes	63	37.3
No	106	62.7
Clavien–Dindo classification		
NA	108	63.9
1–2	40	23.7
3–5	21	12.4
Postoperative hospital stay [days, median (range)]	16	7–208

WDLPS, well-differentiated liposarcoma; DDLPS, dedifferentiated liposarcoma; LMS, leiomyosarcoma; SFT, solitary fibrous tumor; MPNST, malignant peripheral nerve sheath tumor; FNCLCC, The National Federation of Centres for the Fight Against Cancer.

### Disease Recurrence Analysis

**Figure 1:**
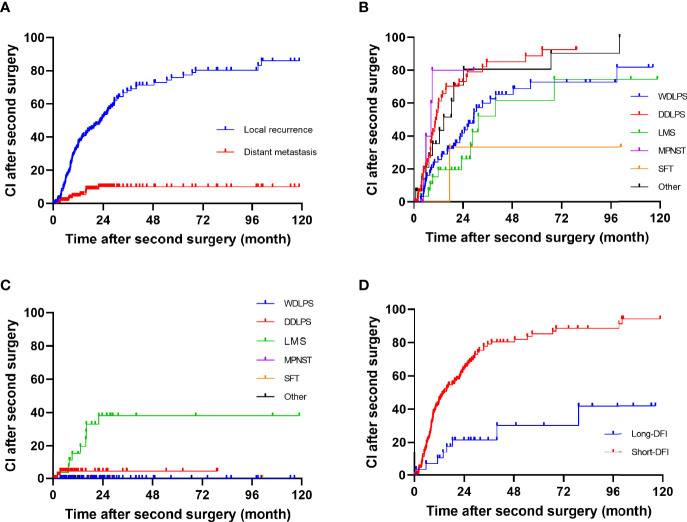
Cumulative incidence in patients after a second surgery for all patients: **(A)** all, **(B)** local recurrence, **(C)** distant metastasis, and **(D)** long disease-free interval group and short disease-free interval group.

The median DFI after second surgery was 19 months (range, 8–57), and the 1-, 2-, and 5-year RFS rate were 62.9% (95% CI, 55.5%–70.3%), 44.8% (95% CI, 36.8%–52.8%), and 23.5% (95% CI, 15.8%–31.7%), respectively. The 1-, 2-, and 5-year crude cumulative incidence for second LR and DM were 34.9% (95% CI, 27.5%–42.43%) and 4.3% (95% CI, 1.0%–7.6%), 51.8% (95% CI, 43.6%–60.0%) and 10.1% (95% CI, 4.2%–16.0%), and 75.9% (95% CI, 67.5%–84.3%) and 10.1% (95% CI, 4.2%–16.0%), respectively (**Figure 1A**).

The univariate analysis for prognostic factors of disease recurrence in patients undergoing resection for first LR is shown in **Table 3**. Disease recurrence was strongly correlated with histologic subtype (*p* = 0.002). The crude cumulative incidence for LR and DM at 5-year for WDLPS, DDLPS, LMS, MPNST, SFT, and others were 72.7% (95% CI, 59.1%–84.3%) and 0% (95% CI, 0%–0%), 88.8% (95% CI, 81.0%–99.6%) and 4.4% (95% CI, 0%–10.5%), 47.7% (95% CI, 24.9%–70.4%) and 38.1% (95% CI, 18.1%–58.1%), NA and NA, 23.3% (95% CI, 0%–76.7%) and NA, and 80.6% (95% CI, 57.1%–100.0%) and 0% (95% CI, 0%–0%), respectively (**Figures 1B, C**). Grade 3 tumors exhibited a greater postoperative recurrence risk (*p* = 0.001) than grades 1–2 tumors. Shorter DFI was also a risk factor for postoperative recurrence. Multifocality, radiation, chemotherapy, high predicted blood loss, longer operative time, and occurrence of postoperative complications were also related to disease recurrence in univariate analysis.

**Table 3:** Univariable and multivariable analyses to determine the independent predictors of disease recurrence.

Variables	Univariate analysis		Multivariate analysis	
	Hazard ratio (95% CI)	*p*-value	Hazard ratio (95% CI)	*p*-value
Gender men vs. women	0.857 (0.578–1.272)	0.444		
Histologic subtypes		0.002		0.326
DDLPS vs. WDLPS	2.242 (1.530–3.841)		1.846 (1.061–3.212)	
LMS vs. WDLPS	1.243 (0.703–2.197)		1.296 (0.674–2.493)	
MPNST vs. WDLPS	3.020 (1.063–8.582)		1.684 (0.538–5.267)	
SFT vs. WDLPS	0.422 (0.058–3.089)		0.447 (0.060–3.318)	
Other vs. WDLPS	2.044 (1.058–3.952)		1.405 (0.688–2.871)	
FNCLCC grade (3 vs. 1–2)	1.953 (0.306 2.921)	0.001	1.122 (0.696–1.807)	0.527
DFI after first surgery (continuous)	0.988 (0.981–0.995)	0.001	0.989 (0.982–0.997)	0.004
Variables at second surgery				
Age (continuous)	0.996 (0.980–1.012)	0.585		
Complete resection (yes vs. no)	0.895 (0.364–2.202)	0.810		
Number of combined resections (continuous)	1.102 (0.964–1.260)	0.154		
Multifocality (yes vs. no)	1.904 (1.260–2.877)	0.002	1.525 (0.956–2.431)	0.076
Radiation (yes vs. no)	0.912 (0.520–1.602)	0.749		
Chemotherapy (yes vs. no)	1.757 (1.126–2.741)	0.013	1.307 (0.787–2.170)	0.301
Operative time (continuous)	1.181 (1.057–1.319)	0.003	1.086 (0.942–1.252)	0.255
Estimated blood loss (continuous)	1.000 (1.000–1.001)	0.008	1.000 (1.000–1.001)	0.621
Packed RBC transfusion (yes vs. no)	1.444 (0.980–2.128)	0.063		
Clavien–Dindo classification (1–5 vs. NA)	1.916 (1.302–2.820)	0.001	1.403 (0.918–2.144)	0.118
Postoperative hospital stay (continuous)	1.005 (0.999–1.010)	0.097		
Variables at first surgery				
Complete resection (yes vs. no)	0.934 (0.636–1.371)	0.728		
Number of combined resections (continuous)	1.097 (0.899–1.340)	0.362		
Radiation (yes vs. no)	0.933 (0.095–9.204)	0.953		
Chemotherapy (yes vs. no)	1.025 (0.998–1.052)	0.075		

WDLPS, well-differentiated liposarcoma; DDLPS, dedifferentiated liposarcoma; LMS, leiomyosarcoma; SFT, solitary fibrous tumor; MPNST, malignant peripheral nerve sheath tumor; FNCLCC, The National Federation of Centres for the Fight Against Cancer; DFI, disease-free interval.

In multivariate analysis, only DFI was an independent risk factor (HR 0.989, *p* = 0.004) for postoperative disease recurrence (**Table 3**). Additionally, decided by the “x-tile” program, we split DFI into long-DFI and short-DFI groups (with a cutoff month of 50) (16). The 5-year cumulative recurrence rate of the long-DFI and short-DFI groups were 47.7% (95% CI, 22.6%–69.8%) and 85.3% (95% CI, 81.5%–93.1%), respectively (**Figure 1D**), with a significant difference between the subgroups (*p* < 0.001).

### Overall Survival Analysis

**Figure 2:**
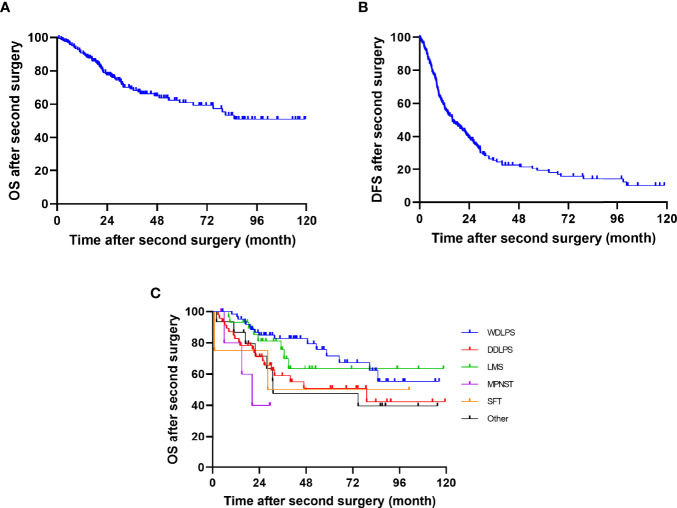
Kaplan–Meier curves of DFS and OS after second surgery of **(A)** OS, **(B)** DFS, and **(C)** OS by histology.

At the most recent checkup, 62 (36.7%) patients were dead throughout the whole group. After removing 5 cases of postoperative deaths, the OS rate at 1, 2, and 5 years was 90.7% (95% CI, 86.2%–95.2.0%), 77.4% (95% CI, 70.7%–84.1%), and 60.9% (95% CI, 51.9%–69.9%), respectively (**Figure 2A**). The 1-, 2-, and 5-year DFS rate were 58.2% (95% CI, 50.8%–65.6%), 40.2% (95% CI, 32.8%–47.6%), and 20.4% (95% CI, 27.8%–13.0%), respectively (**Figure 2B**). The median time from surgery to death was 21.1 (range, 0.8–85.1) months. The 5-year OS for WDLPS, DDLPS, LMS, MPNST, SFT, and others were 71.7% (95% CI, 57.2%–86.2%), 50.9% (95% CI, 33.8%–68.0%), 63.5% (95% CI, 42.2%–84.8%), NA, 50% (95% CI, 4.9%–95.1%), and 47.7% (95% CI, 20.5%–74.9%), respectively (**Figure 2C**).

The univariate analysis results of risk factors for OS are shown in **Table 4**. FNCLCC grade (*p* = 0.008), number of combined resections (*p* < 0.001), operative time (*p* < 0.001), estimated blood loss (*p* < 0.001), packed RBC transfusion (*p* < 0.001), postoperative complication (*p* = 0.003), and chemotherapy at primary surgery (*p* = 0.005) were related to OS in univariate analysis.

**Table 4:** Univariable and multivariable analyses to determine the independent predictors of overall survival.

Variables	Univariate analysis		Multivariate analysis	
	Hazard ratio (95% CI)	*p*-value	Hazard ratio (95% CI)	*p*-value
Gender men vs. women	1.060 (0.813–1.381)	0.668		
Histologic subtypes		0.067		
DDLPS vs. WDLPS	2.148 (1.111–4.151)			
LMS vs. WDLPS	1.235 (0.528–2.887)			
MPNST vs. WDLPS	4.910 (1.404–17.178)			
SFT vs. WDLPS	2.205 (0.507–9.598)			
Other vs. WDLPS	2.212 (0.943–5.191)			
FNCLCC grade (3 vs. 1–2)	2.043 (1.201–3.476)	0.008	2.014 (1.146–3.541)	0.015
DFI after first surgery (continuous)	0.996 (0.988–1.004)	0.376		
Variables at second surgery				
Age (continuous)	1.022 (0.998–1.046)	0.068		
Complete resection (yes vs. no)	1.072 (0.599–1.917)	0.815		
Number of combined resections (continuous)	1.375 (1.151–1.642)	<0.001	1.136 (0.902–1.432)	0.278
Multifocality (yes vs. no)	1.428 (0.808–2.523)	0.220		
Radiation (yes vs. no)	0.950 (0.429–2.104)	0.900		
Chemotherapy (yes vs. no)	1.310 (0.701–2.446)	0.397		
Operative time (continuous)	1.381 (1.191–1.601)	<0.001	1.074 (0.861–1.341)	0.525
Estimated blood loss (continuous)	1.001 (1.000–1.001)	<0.001	1.000 (1.000–1.001)	0.544
Packed RBC transfusion (yes vs. no)	2.725 (1.616–4.594)	<0.001	1.651 (0.789–3.456)	0.184
Clavien–Dindo classification (1–5 vs. NA)	2.203 (1.308–3.711)	0.003	1.310 (0.738–2.327)	0.356
Postoperative hospital stay (continuous)	0.998 (0.988–1.007)	0.639		
Variables at first surgery				
Complete resection (yes vs. no)	0.851 (0.476–1.524)	0.588		
Number of combined resections (continuous)	1.069 (0.789–1.447)	0.669		
Radiation (yes vs. no)	0.962 (0.725–1.277)	0.791		
Chemotherapy (yes vs. no)	1.047 (1.014–1.081)	0.005	1.040 (1.006–1.074)	0.020

WDLPS, well-differentiated liposarcoma; DDLPS, dedifferentiated liposarcoma; LMS, leiomyosarcoma; SFT, solitary fibrous tumor; MPNST, malignant peripheral nerve sheath tumor; FNCLCC, The National Federation of Centres for the Fight Against Cancer; DFI, disease-free interval.

FNCLCC grade, grade 3 vs. grades 1–2 (HR 2.014, *p* = 0.015), and chemotherapy at primary surgery (HR 1.040, *p* = 0.020) were shown to be substantially linked with OS in multivariate Cox analysis.

### Construction and Validation of Nomograms

**Figure 3:**
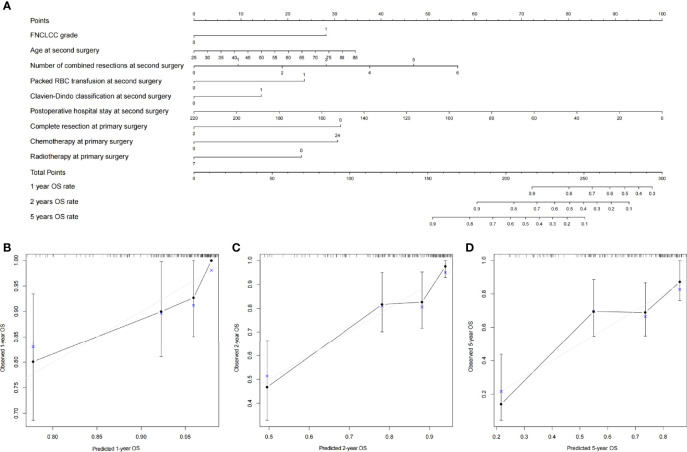
**(A)** Nomogram for 1-, 2-, and 5-year overall survival in patients with first local recurrent retroperitoneal sarcoma and calibration plots for internal validation of **(B)** 1-, **(C)** 2-, and **(D)** 5-year overall survival nomogram.

**Figure 4:**
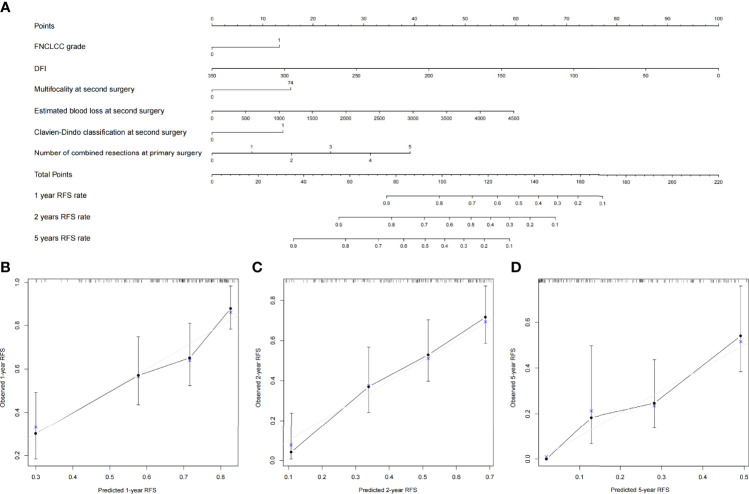
**(A)** Nomogram for 1-, 2-, and 5-year recurrence-free survival in patients with first local recurrent retroperitoneal sarcoma and calibration plots for internal validation of **(B)** 1-, **(C)** 2-, and **(D)** 5-year recurrence-free survival nomogram.

All clinical variables mentioned above were included in the Cox regression analysis, and backward stepwise selection was performed using a likelihood ratio test with Akaike Information Criterion as the stopping criteria. FNCLCC grade, age, number of combined resections, packed RBC transfusion, Clavien–Dindo classification, postoperative hospital stay, complete resection at primary surgery, and chemotherapy/radiotherapy at primary surgery were selected to build the OS nomogram (**Figure 3**), and FNCLCC grade, DFI, multifocality, estimated blood loss, Clavien–Dindo classification, and number of combined resections at primary surgery were chosen to construct the RFS nomogram (**Figure 4**). The calibration plots (**Figures 3**, **4**) demonstrated a high degree of agreement between the nomogram predictions and observed results. The concordance indices were 0.74 (95% CI, 6.1–8.6) for the OS nomogram and 0.70 (95% CI, 0.60–0.79) for the RFS nomogram.

The TARPSWG nomogram was then externally validated using our cohort, and the DFS and OS nomograms had concordance indices of 0.67 and 0.65, respectively.

## Discussion

Soft tissue sarcoma is a rare malignant tumor that accounts for less than 1% of all neoplasm, with around 15% of cases occurring in the retroperitoneal space (17). Surgery is the primary means of curative treatment; however, a large proportion of patients still experience disease recurrence. The mortality rate of RPS is mainly due to LR, and up to 70% of deaths, even when there is no indication of DM (6, 7, 9). Meanwhile, there has been a growth in nomograms being used to predict the prognosis of RPS patients, and several nomograms have been developed to optimize the predictive performance of the disease recurrence and OS of RPS (13, 18, 19). Among them, TARPSWG used multicenter data of European and American populations in 2019 to establish nomogram prediction models for the prognosis of the first relapse of local recurrent retroperitoneal sarcoma. They demonstrated high calibration and the discriminative ability for OS and DFS (C index of 0.70 and 0.67, respectively) (13). In November 2021, Hui et al. performed an external validation of the above nomogram using an Asian cohort of 53 patients; the concordance indices for the 6-year OS was 0.7, but only 0.65 for the 6-year DFS (12), which is consistent with the external validation results in our study (the concordance indices for the 6-year OS and DFS were 0.67 and 0.65, respectively). However, the study of Hui et al. did not investigate the prognostic variables of the Asian population due to the limited sample size. Prognostic factors of FLR-RPS in the Asian population were studied for the first time in this study and found that FNCLCC classification and chemotherapy during the initial surgery were independent prognostic factors of OS and a shorter DFI was linked to postoperative disease recurrence. We established two nomogram prediction models that could successfully predict 1-, 2-, and 5-year OS rate and RFS rate in patients with LR-RPS throughout the same time period.

Both the TARPSWG and our cohorts had comparable and dissimilar prognoses, as seen below. Firstly, the 6-year OS of the TARPSWG set and ours is consistent (54.1% vs. 57.4%), which is significantly higher than the nonsurgical cohort linked to recurrence described in the prior literature (20–22); secondly, the median DFI after the second surgery in the two groups were the same (both 19 months) but is significantly shorter than that of the primary operation [TARPSWG in 2016 reported a median time to LR of primary retroperitoneal tumors after surgery of 39 months (23)]. There are some differences between Asian and Western patients with LR-RPS as well. [1] The 6-year cumulative recurrence rate is higher in our cohort (76.4% vs. 82.4%), and the 6-year cumulative LR is also higher (80.4% vs. 59.0%), but distant metastasis is lower (10.1% vs. 14.6%). First, our cohort had a greater percentage of WDLPS (37.3% vs. 28.1%), and second, the recurrence of WDLPS was frequently local and did not include distant metastasis, both of which are likely contributors to the aforementioned discrepancies (24). Secondly, the proportion of grade 3 in the TARPSWG set was higher (39.4% vs. 29.6%), the risk of distant metastasis of high-grade sarcoma was higher, and the risk of LR was relatively low (23). [2] The Asian cohort (ours and Hui’s) were younger and had a higher tumor burden than the European and American cohorts (TARPSWG cohort).

Our study indicated that DFI was the sole independent prognostic factor for disease recurrence in the examination of risk variables. Specifically, a shorter DFI after primary surgery was associated with a higher risk of recurrence. In FLR-RPS patients undergoing surgery, Yang and colleagues have reported that DFI of less than 1 year was a poor prognostic factor (10). Time to recurrence also exerts significant influence over complete resection rates for recurrent disease (25). DFI’s optimal cutoff value was determined by further using the x-tile software to guide clinical practice. Within 5 years, 85.3% of patients in the short-DFI group experienced disease recurrence, compared with just 47.7% of patients in the long-DFI group when the cutoff value was 50 months. Therefore, the interval between follow-up following second surgery should be decreased if the duration from initial resection to disease recurrence is shorter than 4 years.

In the multivariate analysis of OS-related factors, FNCLCC grade was revealed as an independent prognostic parameter. The degree of tumor differentiation, rate of mitosis, and degree of necrosis are the three primary variables for the globally accepted FNCLCC classification. Various factors, such as mitosis rate, degree of necrosis, and degree of tumor differentiation, are considered by clinicians when determining the grade based on the sum of the scores. Twofold (*p* = 0.015) greater risk of mortality was seen for high-grade sarcoma compared with low-grade FNCLCC in this analysis, which is consistent with the conclusions from prior studies reporting on primary or recurrent RPS (13, 22). A worse survival following second surgery for LR was also connected with initial surgical chemotherapy, as we discovered. This should not be interpreted as chemotherapy influencing the long-term survival of patients. Rather, it is more probable that patients who undergo chemotherapy have aggressive tumor biology. Patients with “high-risk” tumors are more likely to be given chemotherapy, according to a recent trend. Chemotherapy’s effect on RPS has yet to be determined. There is no conclusive evidence that preventive systemic therapy has an impact following full resection of recurrent RPS (26–28).

There are several limitations to this study. First, due to its retrospective nature, it is susceptible to subject selection bias. A second issue is that we were unable to confirm uncommon histological subtypes, such as MPNST (*n* = 6), due to the cohort’s low representation. Thirdly, although the performance of this nomogram was shown to be beneficial in our cohort, external validation is still necessary to be sure.

This study, which was the first to investigate the prognostic characteristics of patients with RPS for the first LR with the largest monocentric Asian experience, discovered that the postoperative DFI was associated with recurrence of the disease after LR; the initial surgical chemotherapy and high-grade tumors were associated with OS. In addition, two nomogram prediction models concerning OS and RFS, which can reliably predict the prognosis of FLR-RPS patients, were developed by our team.

## Data Availability Statement

The raw data supporting the conclusions of this article will be made available by the authors, without undue reservation.

## Ethics Statement

The studies involving human participants were reviewed and approved by the ethics committee of the South Hospital of Zhongshan Hospital/Shanghai Public Health Clinical Center. The patients/participants provided their written informed consent to participate in this study.

## Author Contributions

Conceptualization: AZ and WL. Methodology: AZ and LM. Software: AZ and YF. Validation: JW and JX. Formal analysis: AZ and JX. Investigation: AZ. Resources: YZ. Data curation: JW. Writing—original draft preparation: AZ. Writing—review and editing: LM. Visualization: YF. Supervision: HT. Project administration: AZ. Funding acquisition: YZ. All authors have read and agreed to the published version of the manuscript.

## Conflict of Interest

The authors declare that the research was conducted in the absence of any commercial or financial relationships that could be construed as a potential conflict of interest.

## Publisher’s Note

All claims expressed in this article are solely those of the authors and do not necessarily represent those of their affiliated organizations, or those of the publisher, the editors and the reviewers. Any product that may be evaluated in this article, or claim that may be made by its manufacturer, is not guaranteed or endorsed by the publisher.
